# Editorial: Targeting redox regulation and autophagy systems for cancer therapy

**DOI:** 10.3389/fonc.2023.1146670

**Published:** 2023-01-31

**Authors:** Junmin Zhang, Abdelhabib Semlali, Paola Maycotte, Luciano Saso

**Affiliations:** ^1^ School of Pharmacy and State Key Laboratory of Applied Organic Chemistry, Lanzhou University, Lanzhou, China; ^2^ Groupe de Recherche en Écologie Buccale, Faculté de Médecine Dentaire, Université Laval, Quebec, QC, Canada; ^3^ Centro de Investigación Biomédica de Oriente, Instituto Mexicano del Seguro Social, Puebla, Mexico; ^4^ Department of Physiology and Pharmacology "Vittorio Erspamer", Sapienza University of Rome, Rome, Italy

**Keywords:** redox regulation, reactive oxygen species, oxidative stress, redox-active compounds, autophagy, apoptosis, ferroptosis, cancer therapy

Autophagy and increased ROS production are important contributors to cancer metabolic remodeling (1, 2), and an interplay between both processes has been described with important consequences on cancer cell survival and death (3). Both ROS production and autophagy have a context and stage-dependent role on tumorigenesis and cancer progression. For instance, autophagy is a tumor suppressor pathway but can promote cancer cell survival under diverse stress conditions (4). Similarly, increased ROS have been implicated in tumorigenesis caused by diverse infectious or environmental agents as well as in the maintenance of cancer cell signaling pathways (5, 6), but have been suggested to limit metastasis formation (7, 8). Thus, a detailed study of the role of ROS and autophagy is needed, to understand their role in cancer initiation and progression and to design effective therapies targeting these mechanisms.

This issue includes manuscripts covering diverse aspects of ROS and autophagy in cancer progression and therapy. The review by Hasan et al., describes the close interplay between ROS and autophagy in cancer cells and the complexity involved in designing anticancer therapies targeting these processes. In a different review, Roy et al. describe the diverse roles of autophagy in response to cancer radiotherapy (RT), outlining the role of autophagy in deciding the cellular fate upon exposure to radiation, in cancer stem cell (CSC) maintenance and the radiation-induced bystander effect as well as its contribution to cell survival or death and its interplay with radiation-induced ROS. The authors emphasize the role of autophagy inhibitors and inducers in radiosensitization and, they refer to studies where autophagy markers were related to a poor therapeutic outcome in patients treated with RT or chemo-radiotherapy (CRT). The authors also mention clinical trials using autophagy inhibitors as adjuvant to CRT in glioblastoma with promising results as well as another one in pancreatic cancer patients where no significant improvement in survival was observed. They conclude that an encouraging clinical response using autophagy regulators is needed to incorporate them as adjuvant to current RT or CRT regimens.

Two interesting manuscripts describe gene signatures related to autophagy modulation. The first one, by Wu et al. describes a gene signature with prognostic significance consisting of genes participating in autophagy in lung adenocarcinoma (LUAD), with functions in immune and tumor-related pathways. The second manuscript by Liu et al., describes a gene signature consisting of three autophagy-related long noncoding RNAs (lncRNAs) in cholangiocarcinoma (CCA), with prognostic potential, and whose lncRNAs were found to be increased in CCA tissue when compared to normal tissue. Importantly, in both papers, the authors found differences in the enrichment scores for immune cells between low and high-risk groups, as well as different expression levels of checkpoint inhibitors in Wu et al., suggesting an important role for the expression of genes participating in autophagy, or regulating autophagy in the definition of the immune landscape in LUAD and CCA. Another interesting paper using data analysis is the one by Zhu et al. In this manuscript, the authors analyzed BNIP3, an apoptosis regulator and mitophagy receptor in scRNA-seq datasets, identifying cancer cell subpopulations characterized by high BNIP3 levels in most epithelial malignancies characterized by NRF2 signaling, HIF1A, wound response, metabolic reprogramming, high ROS-related pathways, oxidative phosphorylation and MYC targets. Also, high BNIP3 mRNA was a worse prognostic factor for cervical squamous cell carcinoma, endocervical adenocarcinoma, cholangiocarcinoma, sarcoma and breast cancer and BNIP3 was overexpressed in liver cancer organoids. This study emphasizes the role of the BNIP3-high cellular population in defining the prognosis of certain cancer types. Also relating to autophagy and cancer therapy, the manuscript by Karim et al., describes a phase I clinical trial to determine the maximum tolerated dose (MTD) of chloroquine (CQ) or hydroxychloroquine (HCQ), two of the most widely used autophagy inhibitors, in combination with carboplatin and gemcitabine in heavily pre-treated patients with advanced solid tumors. The authors found a MTD of HCQ of 100 mg when given in combination with carboplatin and gemcitabine, a lower than previously reported MTDs, probably due to the myelosuppressive nature of gemcitabine. The overall response rate was 71% and limiting factors were neutropenia and thrombocytopenia. The authors also observed that patients receiving subsequent immunotherapy after participating in this clinical trial had excellent clinical outcomes, suggesting a promising response, and warranting progression to a phase II clinical trial.

Regarding potential therapeutic interventions for different cancer types, Zhu et al. describe the use of elaiophylin, a macrodiolide antibiotic isolated from *Streptomyces melanosporus* which can function as a late-stage autophagy inhibitor in uveal melanoma cell lines. The authors found that elaiophylin induced cell death by inducing oxidative stress, mitochondrial dysfunction, regulating SIRT1, FoxO3a, and decreasing mitophagy. Similar results were found in a xenograft model, implying the use of elaiophylin for the treatment of this rare, but deadly cancer. Another study using a natural compound is the one by Alsamri et al., where the authors use carnosol, a phenolic compound against breast cancer cell lines. The authors found that carnosol triggered a ROS-dependent endoplasmic reticulum (ER)-stress response, p38-dependent autophagy, activation of ATF6 and IRE-1a and mTOR degradation in the proteasome. This study adds up carnosol to natural compounds known to trigger a ROS-dependent ER-stress mediated cell death emphasizing its potential use for breast cancer therapy. Regarding differentiation therapies, the manuscript by Benjamin et al. describes the use of valproic acid (VPA), an autophagy inducer, in all-*trans*-retinoic acid (ATRA) resistant acute promyelocytic leukemia (APL), a type of acute myeloid leukemia (AML). The authors describe a key role for autophagy in the differentiation of APL cells induced by ATRA and VPA combination treatment and implying a potential use for autophagy inducers in this type of therapy. Another therapeutic intervention is presented in the manuscript by Semlali et al., which describes the use of rapamycin for oral cancer treatment. Rapamycin induced cell death in oral cancer cells when compared to controls involving total ROS and mitochondrial ROS production, DNA damage, MAPK, NFkB and Wnt/b-catenin pathway inhibition. Interestingly, antioxidants in combination with an autophagy inhibitor decreased cancer cell death suggesting both mechanisms participate in cell death induction by rapamycin. Another study by Park et al. describes an association between nuclear factor erythroid 2-like 2 (NRF2), a master transcriptional factor for antioxidant response genes and the CSC marker CD133 in colon cancer stem cells. The authors found that CD133 controls NRF2 expression levels by regulating Akt and GSK-3β phosphorylation. These results support an important role for ROS detoxifying mechanisms in supporting the aggressive phenotype of CSCs. Finally, the study by Ling et al., describes the use of a superoxide dismutase 1 (SOD1) inhibitor (LCS-1) for glioma treatment. The authors found increased SOD1 levels in grade III and IV gliomas as well as SOD1/2 expression in glioma cell lines, where LCS-1 induced ROS-dependent, p53- and caspase-independent cell death with PARP and BRCA1/2 degradation. LCS-1 also decreased tumor growth in a mouse xenograft model highlighting the potential use of LCS-1 or ROS-inducing agents for the treatment of this deadly disease.

Altogether, this collection of manuscripts covers aspects of the ROS-autophagy-cancer interplay, discussing novel therapeutic targets for different cancer types. We hope you enjoy them.

## Author contributions

All authors listed have made a substantial, direct, and intellectual contribution to the work and approved it for publication.
